# Role of Ras isoforms in γδ T-cell development

**DOI:** 10.1186/1479-5876-10-S3-O6

**Published:** 2012-11-28

**Authors:** Ana V Marín-Marín, Eva Jiménez Pérez, Eugenio Santos, Edgar Fernández-Malavé

**Affiliations:** 1 Dept. of Immunology, Faculty of Medicine, Complutense University, Madrid, Spain; 2 Cellular Biology Dept., Faculty of Medicine, Complutense University, Madrid, Spain; 3 Center for Cancer Research, Salamanca, Spain

## Introduction

The small guanine nucleotide binding proteins of the Ras family (H-, K-, and N-ras isoforms) couple surface receptors, including T-cell antigen receptors, to a variety of cellular responses. Ras proteins are highly homologous and expressed ubiquitously, raising questions as to their functional specificity. H-ras and N-ras have been shown to be dispensable for development in the thymus but critical for peripheral Th1 differentiation of αβ T cells [1]. In the γδ T-cell lineage, in contrast, the specific function of Ras isoforms had not been addressed.

## Aim

We aimed at identifying the specific roles of H-ras and N-ras in γδ T-cell development, particularly, in the generation of γδ T-cell subsets defined by expression of CD27.

## Mice and methods

Mice deficient for H-ras or N-ras were analyzed by multi-parametric flow cytometry. In addition, N-ras-deficient mice with impaired αβ T-cell development were generated by breeding with CD3δ KO mice and analyzed.

## Results

Ras isoform-deficient mice exhibited normal frequencies and numbers of γδ T cells in the thymus, which expressed normal levels of surface γδTCR, but a consistent decrease of CD27^+^ γδ T cells in peripheral lymphoid organs, compared to controls. Conversely, in mice lacking mature αβ T cells, N-ras deficiency resulted in reduced numbers of CD27^+^ γδ T cells, with concomitant increase of the CD27^-^ population, in the thymus but not in the periphery. This suggests that signals from αβ T-cell thymocytes for developing CD27^+^ γδ T cells are partly N-ras-dependent.

## Conclusions

As previously shown for αβ T cells, H-ras and N-ras are dispensable for intrathymic development of γδ T cells, but they could be involved in finely regulating survival and/or expansion of γδ T cells in the periphery, in agreement with recent findings in mice lacking RasGRP1 [2], a guanine nucleotide exchange factor for Ras.

## Acknowledgements

The study was supported by grant FIS PI11/02198 from Instituto de Salud Carlos III to EF-M.
